# Supplementary material for: A Likelihood Approach for Real-Time Calibration of Stochastic Compartmental Epidemic Models
Source: PLoS Comput Biol. 2017 Jan 17;13(1):e1005257. doi: 10.1371/journal.pcbi.1005257 (PMC5240920; doi:10.1371/journal.pcbi.1005257)
Supplement: S1 File — (TAR.GZ) [file pcbi.1005257.s014.tar.gz › HSPH_Online-SI-Revision/Additions/IPoi10/n100-extreme-SEITR_noisy/n100-extreme-SEITR_noisy_BENCH_table4.pdf]

1	R0	{1.38654, 9.85565}	{2.86095, 19.9929}	{{2.86095, 19.9929}, {2.86095, 19.9929}}	{106.337, 102.857}	inf	602.	17110.3	{9886., 25535.}	259.611	0.530165	newly	2726.36	5483.86	{3062., 8526.}	101.877	0.276769	Reff	0.128602	0.466912	{0.293305, 0.684768}	263.069	ar	92379.	93010.6	{92875., 93128.}	0.683666
2	R0	{2.36629, 12.2029}	{2.9884, 12.8326}	{{2.9884, 12.8326}, {2.9884, 12.8326}}	{26.2905, 5.16025}	inf	104.	13122.4	{6868., 23886.}	123.314	0.304211	newly	2664.	6135.6	{3079., 11331.}	131.038	0.310104	Reff	0.0301229	0.35256	{0.232258, 0.53322}	1070.41	ar	99022.	93965.8	{93794., 94108.}	5.1061
3	R0	{2.32253, 12.836}	{2.9194, 10.1535}	{{2.9194, 10.1535}, {2.9194, 10.1535}}	{25.6991, 20.8983}	inf	185.	5916.88	{2945., 12135.}	44.3909	0.245869	newly	3413.	3174.68	{1561., 6599.}	44.4405	0.207217	Reff	0.035767	0.24102	{0.211189, 0.307822}	573.863	ar	98997.	93462.	{93311., 93603.}	5.59103
4	R0	{1.79075, 11.2058}	{2.9884, 12.8326}	{{2.9884, 12.8326}, {2.9884, 12.8326}}	{66.8798, 14.5175}	inf	424.	9412.7	{4648., 17310.}	58.1808	0.1766	newly	3304.	4273.24	{2056., 7987.}	55.1223	0.180587	Reff	0.0739401	0.272472	{0.212356, 0.364226}	268.504	ar	96920.	93950.9	{93820., 94094.}	3.06347
5	R0	{2.26952, 9.62371}	{2.98388, 9.60029}	{{2.98388, 9.60029}, {2.98388, 9.60029}}	{31.4763, 0.243357}	inf	39.	5223.91	{2437., 9422.}	113.03	0.271483	newly	1318.	2933.69	{1405., 5339.}	124.646	0.289833	Reff	0.0289591	0.223276	{0.197891, 0.263119}	671.006	ar	98881.	93953.1	{93794., 94076.}	4.98369
6	R0	{1.57191, 6.89004}	{2.91001, 10.5991}	{{2.91001, 10.5991}, {2.91001, 10.5991}}	{85.1257, 53.8322}	inf	100.	7247.57	{3401., 11692.}	399.488	0.625796	newly	1057.	3857.14	{1784., 6207.}	264.914	0.482139	Reff	0.0825567	0.265553	{0.218396, 0.303892}	221.662	ar	95046.	93396.5	{93244., 93552.}	1.73551
7	R0	{1.99226, 17.2339}	{2.97157, 19.3547}	{{2.97157, 19.3547}, {2.97157, 19.3547}}	{49.1557, 12.306}	inf	602.	11408.9	{6659., 18143.}	30.004	0.133629	newly	3977.	3677.23	{2119., 5947.}	31.6088	0.151225	Reff	0.0686533	0.329386	{0.237785, 0.385383}	379.782	ar	97992.	93832.	{93626., 93966.}	4.24529
8	R0	{2.63369, 15.1879}	{2.9884, 12.8326}	{{2.9884, 12.8326}, {2.9884, 12.8326}}	{13.4682, 15.5077}	inf	100.	11123.9	{7202., 18279.}	41.6777	0.136381	newly	3068.	5093.34	{3183., 8425.}	67.9669	0.198363	Reff	0.022123	0.30451	{0.230884, 0.390405}	1276.44	ar	99451.	93956.1	{93790., 94121.}	5.52518
9	R0	{2.53271, 9.90552}	{2.98388, 9.60029}	{{2.98388, 9.60029}, {2.98388, 9.60029}}	{17.8137, 3.08141}	inf	27.	5129.05	{2477., 9237.}	102.167	0.246909	newly	1330.	2867.82	{1386., 5198.}	117.491	0.272447	Reff	0.0191979	0.222151	{0.197742, 0.255719}	1057.16	ar	99355.	93944.8	{93783., 94082.}	5.44529
10	R0	{2.48791, 18.9003}	{2.7943, 13.5962}	{{2.7943, 13.5962}, {2.7943, 13.5962}}	{12.3152, 28.0636}	inf	387.	6641.96	{3087., 11294.}	59.4128	0.42501	newly	4707.	2876.16	{1297., 4856.}	50.3477	0.311211	Reff	0.0381148	0.276142	{0.238717, 0.32738}	624.5	ar	99291.	92441.2	{92312., 92581.}	6.89869
11	R0	{2.84414, 18.4765}	{2.86118, 15.5722}	{{2.86118, 15.5722}, {2.86118, 15.5722}}	{0.599127, 15.7189}	inf	161.	12447.4	{6997., 18505.}	26.9913	0.129258	newly	4255.	4845.51	{2634., 7269.}	34.3231	0.13512	Reff	0.0214164	0.333761	{0.262141, 0.406373}	1458.44	ar	99654.	93015.	{92817., 93199.}	6.66204
12	R0	{2.29369, 14.622}	{2.7943, 13.5962}	{{2.7943, 13.5962}, {2.7943, 13.5962}}	{21.8255, 7.01546}	inf	189.	12295.3	{6624., 20851.}	64.4987	0.192757	newly	3139.	5415.7	{2862., 9343.}	78.4579	0.217945	Reff	0.0347723	0.35928	{0.271606, 0.509038}	933.234	ar	98982.	92435.1	{92266., 92601.}	6.61427
13	R0	{2.73413, 16.7529}	{2.99939, 13.7076}	{{2.99939, 13.7076}, {2.99939, 13.7076}}	{9.70181, 18.1777}	inf	128.	8892.76	{4601., 15776.}	39.7578	0.195936	newly	3594.	3792.56	{1908., 6737.}	40.994	0.166797	Reff	0.0212989	0.2588	{0.210797, 0.338031}	1115.09	ar	99543.	94066.4	{93892., 94224.}	5.50172
14	R0	{1.79554, 16.5943}	{2.76795, 19.6041}	{{2.76795, 19.6041}, {2.76795, 19.6041}}	{54.157, 18.1376}	inf	1182.	11145.7	{5809., 18022.}	32.8071	0.173797	newly	4850.	3609.27	{1778., 5839.}	37.5544	0.211054	Reff	0.106063	0.377519	{0.268076, 0.434513}	255.94	ar	96821.	92212.4	{92071., 92338.}	4.7599
15	R0	{1.91429, 13.7771}	{2.93978, 19.9817}	{{2.93978, 19.9817}, {2.93978, 19.9817}}	{53.5703, 45.0356}	inf	383.	13772.5	{7799., 21640.}	72.586	0.212941	newly	3380.	4393.76	{2393., 6844.}	42.5626	0.143495	Reff	0.0667896	0.396239	{0.251351, 0.518783}	493.265	ar	97541.	93620.8	{93473., 93784.}	4.01907
16	R0	{1.42094, 10.5871}	{2.8939, 16.8013}	{{2.8939, 16.8013}, {2.8939, 16.8013}}	{103.661, 58.696}	inf	1054.	11238.3	{6050., 22057.}	60.3217	0.183737	newly	4015.	4086.98	{2153., 8269.}	36.3818	0.157279	Reff	0.134932	0.314052	{0.23866, 0.504436}	132.748	ar	93044.	93265.6	{93139., 93411.}	0.244298
17	R0	{1.45515, 6.16466}	{2.9194, 10.1535}	{{2.9194, 10.1535}, {2.9194, 10.1535}}	{100.625, 64.7049}	inf	170.	8034.02	{4051., 12528.}	363.056	0.614594	newly	1376.	4370.51	{2193., 6771.}	217.624	0.446661	Reff	0.105091	0.266296	{0.219393, 0.307822}	153.396	ar	93254.	93460.7	{93324., 93599.}	0.222057
18	R0	{2.85669, 13.2416}	{2.83858, 10.6973}	{{2.83858, 10.6973}, {2.83858, 10.6973}}	{0.63395, 19.2144}	inf	31.	7899.43	{4341., 12895.}	45.0823	0.156156	newly	2583.	4122.53	{2196., 6810.}	65.922	0.194746	Reff	0.014512	0.275587	{0.235091, 0.332029}	1799.03	ar	99644.	92849.3	{92710., 93007.}	6.81894
19	R0	{2.56417, 17.9558}	{2.8939, 16.8013}	{{2.8939, 16.8013}, {2.8939, 16.8013}}	{12.8591, 6.42968}	inf	266.	17153.7	{10751., 24797.}	35.1856	0.128676	newly	4524.	6354.83	{3895., 9339.}	47.0563	0.156208	Reff	0.0335137	0.419416	{0.289477, 0.615098}	1151.48	ar	99389.	93275.	{93107., 93466.}	6.15159
20	R0	{2.27517, 11.1864}	{2.92375, 10.9639}	{{2.92375, 10.9639}, {2.92375, 10.9639}}	{28.5069, 1.98902}	inf	92.	8519.98	{4834., 13475.}	97.8448	0.262406	newly	2013.	4445.37	{2456., 6995.}	121.139	0.301598	Reff	0.0313746	0.289557	{0.229894, 0.326349}	822.904	ar	98894.	93500.4	{93362., 93647.}	5.45397
21	R0	{2.35735, 10.4735}	{2.9194, 10.1535}	{{2.9194, 10.1535}, {2.9194, 10.1535}}	{23.8425, 3.05533}	inf	65.	9135.66	{4494., 16471.}	146.343	0.328463	newly	1848.	5135.65	{2377., 9194.}	178.377	0.36735	Reff	0.0275103	0.305241	{0.225436, 0.377858}	1009.55	ar	99036.	93489.8	{93310., 93639.}	5.6002
22	R0	{1.79551, 11.9179}	{2.7943, 13.5962}	{{2.7943, 13.5962}, {2.7943, 13.5962}}	{55.6271, 14.0822}	inf	355.	7683.17	{3528., 14167.}	57.7741	0.203051	newly	2882.	3355.85	{1519., 6186.}	57.5771	0.21443	Reff	0.0710663	0.29555	{0.237013, 0.365075}	315.88	ar	97033.	92459.8	{92223., 92649.}	4.71307
23	R0	{1.23545, 6.31687}	{2.9884, 12.8326}	{{2.9884, 12.8326}, {2.9884, 12.8326}}	{141.888, 103.148}	inf	264.	8296.29	{3737., 15995.}	400.078	0.645193	newly	1370.	3729.05	{1632., 7394.}	172.193	0.378233	Reff	0.148353	0.253121	{0.206678, 0.336075}	70.6206	ar	88739.	93958.9	{93811., 94115.}	5.8823
24	R0	{2.24089, 10.1789}	{2.83858, 10.6973}	{{2.83858, 10.6973}, {2.83858, 10.6973}}	{26.672, 5.09289}	inf	88.	9362.62	{4443., 17265.}	124.764	0.297189	newly	2182.	5018.1	{2295., 9238.}	131.72	0.302409	Reff	0.0325825	0.313669	{0.237561, 0.406258}	862.69	ar	98868.	92843.6	{92682., 92975.}	6.09336
25	R0	{1.57298, 10.2607}	{2.86118, 15.5722}	{{2.86118, 15.5722}, {2.86118, 15.5722}}	{81.8955, 51.7655}	inf	633.	15085.9	{9201., 22443.}	130.002	0.337291	newly	3489.	5956.92	{3523., 9017.}	71.6505	0.208228	Reff	0.10572	0.392903	{0.28277, 0.521593}	271.645	ar	94957.	93006.2	{92855., 93138.}	2.05444
26	R0	{1.2154, 8.2576}	{2.8939, 16.8013}	{{2.8939, 16.8013}, {2.8939, 16.8013}}	{138.103, 103.465}	inf	684.	13808.7	{7945., 22922.}	256.354	0.522455	newly	2648.	5102.27	{2809., 8818.}	93.1979	0.253292	Reff	0.165416	0.379795	{0.259756, 0.53378}	129.6	ar	88421.	93279.8	{93160., 93390.}	5.49512
27	R0	{2.22841, 18.7983}	{2.8939, 16.8013}	{{2.8939, 16.8013}, {2.8939, 16.8013}}	{29.8639, 10.6233}	inf	454.	7441.22	{3964., 13060.}	46.8894	0.301924	newly	4125.	2668.4	{1397., 4682.}	43.2427	0.264009	Reff	0.0518997	0.262006	{0.222888, 0.320239}	404.832	ar	98756.	93249.7	{93077., 93406.}	5.57563
28	R0	{1.27778, 5.36767}	{2.9194, 10.1535}	{{2.9194, 10.1535}, {2.9194, 10.1535}}	{128.474, 89.1603}	inf	160.	5388.13	{2648., 9775.}	384.109	0.62985	newly	965.	2894.97	{1398., 5317.}	199.997	0.41983	Reff	0.136275	0.235758	{0.20973, 0.269753}	73.0016	ar	89843.	93448.4	{93354., 93581.}	4.01305
29	R0	{2.38042, 15.8901}	{2.99939, 13.7076}	{{2.99939, 13.7076}, {2.99939, 13.7076}}	{26.0026, 13.735}	inf	266.	6680.97	{3055., 10205.}	45.5613	0.300078	newly	4278.	2842.69	{1301., 4416.}	39.6154	0.24718	Reff	0.0399673	0.231726	{0.199759, 0.267606}	479.789	ar	99112.	94038.9	{93890., 94148.}	5.11851
30	R0	{2.53188, 12.7607}	{2.9194, 10.1535}	{{2.9194, 10.1535}, {2.9194, 10.1535}}	{15.3056, 20.4315}	inf	140.	8455.69	{4468., 13648.}	41.5571	0.171878	newly	3519.	4613.02	{2403., 7471.}	52.0793	0.170104	Reff	0.0262556	0.272805	{0.224006, 0.324988}	939.038	ar	99317.	93451.2	{93301., 93570.}	5.90613
31	R0	{1.24482, 10.5471}	{2.86095, 19.9929}	{{2.86095, 19.9929}, {2.86095, 19.9929}}	{129.828, 89.5583}	inf	1178.	13157.8	{8385., 19755.}	119.442	0.315091	newly	3288.	4154.62	{2572., 6268.}	39.1391	0.134947	Reff	0.174511	0.365466	{0.268758, 0.443304}	109.422	ar	89097.	93003.7	{92839., 93187.}	4.38478
32	R0	{2.30446, 17.9607}	{2.7943, 13.5962}	{{2.7943, 13.5962}, {2.7943, 13.5962}}	{21.2562, 24.3003}	inf	386.	7994.87	{4441., 12629.}	45.573	0.280474	newly	4308.	3454.75	{1901., 5474.}	38.2268	0.198224	Reff	0.0453748	0.290032	{0.247715, 0.34096}	539.192	ar	99028.	92450.	{92289., 92636.}	6.64252
33	R0	{2.31579, 11.8236}	{2.98388, 9.60029}	{{2.98388, 9.60029}, {2.98388, 9.60029}}	{28.8493, 18.804}	inf	79.	1923.05	{820., 3736.}	65.5562	0.490916	newly	2012.	1066.85	{453., 2082.}	62.6308	0.427164	Reff	0.0280905	0.194603	{0.184434, 0.206932}	592.77	ar	99012.	93951.2	{93824., 94077.}	5.11132
34	R0	{1.6678, 10.6744}	{2.96307, 14.3219}	{{2.96307, 14.3219}, {2.96307, 14.3219}}	{77.6634, 34.1705}	inf	325.	8816.33	{4559., 18824.}	90.3954	0.223252	newly	2446.	3642.87	{1867., 7898.}	62.2118	0.172388	Reff	0.0850078	0.270577	{0.217845, 0.404726}	218.297	ar	95809.	93797.	{93652., 93916.}	2.1
35	R0	{1.65476, 7.07559}	{2.9194, 10.1535}	{{2.9194, 10.1535}, {2.9194, 10.1535}}	{76.4244, 43.5004}	inf	106.	7550.47	{4310., 12089.}	347.568	0.620546	newly	1151.	4066.23	{2350., 6632.}	253.278	0.516478	Reff	0.0754074	0.256044	{0.224093, 0.305048}	239.547	ar	95746.	93470.5	{93335., 93636.}	2.37661
36	R0	{2.71807, 18.5959}	{2.95376, 15.278}	{{2.95376, 15.278}, {2.95376, 15.278}}	{8.67123, 17.8421}	inf	279.	15765.7	{10546., 22623.}	24.7681	0.105855	newly	5029.	6338.1	{4116., 9259.}	35.4371	0.122874	Reff	0.0291921	0.408878	{0.27786, 0.498477}	1300.65	ar	99540.	93730.3	{93566., 93871.}	5.8366
37	R0	{2.6202, 12.7552}	{2.99939, 13.7076}	{{2.99939, 13.7076}, {2.99939, 13.7076}}	{14.4718, 7.46676}	inf	74.	11528.7	{6737., 18143.}	73.4114	0.207313	newly	2840.	4971.04	{2841., 7743.}	76.276	0.211143	Reff	0.0220097	0.29395	{0.228344, 0.369285}	1235.55	ar	99426.	94028.1	{93863., 94203.}	5.42907
38	R0	{1.47672, 9.81651}	{2.86118, 15.5722}	{{2.86118, 15.5722}, {2.86118, 15.5722}}	{93.7524, 58.6328}	inf	503.	9560.39	{5269., 16211.}	106.594	0.269807	newly	2643.	3711.81	{2017., 6388.}	54.7884	0.166894	Reff	0.109425	0.303269	{0.241226, 0.386603}	177.148	ar	93815.	92999.8	{92876., 93141.}	0.868901
39	R0	{1.90004, 12.3313}	{2.7943, 13.5962}	{{2.7943, 13.5962}, {2.7943, 13.5962}}	{47.0653, 10.2576}	inf	369.	8870.74	{4755., 13067.}	41.9578	0.149993	newly	3317.	3902.13	{2215., 5670.}	40.6495	0.149232	Reff	0.0681354	0.341602	{0.253471, 0.361471}	401.357	ar	97413.	92434.4	{92270., 92624.}	5.11081
40	R0	{2.64218, 18.5237}	{2.9884, 12.8326}	{{2.9884, 12.8326}, {2.9884, 12.8326}}	{13.1036, 30.7233}	inf	174.	3501.57	{1890., 6617.}	69.8893	0.56482	newly	3674.	1552.6	{830., 2890.}	57.8447	0.418845	Reff	0.0267917	0.205695	{0.190929, 0.23447}	667.755	ar	99474.	93973.7	{93824., 94149.}	5.5294
41	R0	{1.6945, 7.17996}	{2.9194, 10.1535}	{{2.9194, 10.1535}, {2.9194, 10.1535}}	{72.2868, 41.4144}	inf	141.	8552.32	{4352., 12738.}	243.467	0.484547	newly	1635.	4777.93	{2323., 6987.}	192.228	0.40283	Reff	0.0694067	0.30337	{0.223159, 0.317251}	337.091	ar	96332.	93477.3	{93323., 93636.}	2.96345
42	R0	{1.84559, 18.0074}	{2.97157, 19.3547}	{{2.97157, 19.3547}, {2.97157, 19.3547}}	{61.0092, 7.48192}	inf	1048.	12312.2	{7154., 21748.}	33.5395	0.160405	newly	4650.	3975.78	{2263., 7096.}	35.6864	0.177473	Reff	0.093313	0.317994	{0.236626, 0.458305}	240.782	ar	97358.	93862.5	{93713., 93996.}	3.59033
43	R0	{2.24965, 17.0361}	{2.7943, 13.5962}	{{2.7943, 13.5962}, {2.7943, 13.5962}}	{24.2104, 20.1918}	inf	520.	5988.24	{2989., 9866.}	56.4035	0.404278	newly	4598.	2565.37	{1293., 4207.}	49.1921	0.322618	Reff	0.0537891	0.265411	{0.233548, 0.312067}	393.428	ar	98770.	92453.3	{92271., 92626.}	6.39537
44	R0	{2.54324, 15.7967}	{2.7943, 13.5962}	{{2.7943, 13.5962}, {2.7943, 13.5962}}	{9.87166, 13.9301}	inf	127.	12414.6	{6918., 22288.}	50.4748	0.162029	newly	3246.	5474.22	{2879., 10138.}	72.8701	0.206277	Reff	0.0255087	0.361427	{0.273925, 0.575682}	1316.88	ar	99354.	92450.7	{92242., 92649.}	6.94821
45	R0	{2.54943, 14.8032}	{2.9884, 12.8326}	{{2.9884, 12.8326}, {2.9884, 12.8326}}	{17.2184, 13.312}	inf	120.	12106.8	{6879., 18749.}	55.0444	0.175399	newly	3187.	5502.71	{3089., 8586.}	75.2342	0.21873	Reff	0.0250609	0.296681	{0.232408, 0.381051}	1083.84	ar	99380.	93970.5	{93804., 94115.}	5.4432
46	R0	{2.37755, 12.6461}	{2.9194, 10.1535}	{{2.9194, 10.1535}, {2.9194, 10.1535}}	{22.7903, 19.7104}	inf	129.	2980.28	{1483., 4755.}	60.7184	0.44738	newly	3124.	1590.32	{771., 2521.}	53.8874	0.366141	Reff	0.0309795	0.212984	{0.19884, 0.229144}	587.502	ar	99096.	93478.7	{93344., 93617.}	5.66851
47	R0	{1.69279, 11.6636}	{2.95376, 15.278}	{{2.95376, 15.278}, {2.95376, 15.278}}	{74.4906, 30.9887}	inf	535.	8896.9	{4609., 13605.}	40.4442	0.144706	newly	3657.	3512.51	{1794., 5394.}	36.4849	0.160124	Reff	0.0899041	0.277837	{0.22041, 0.323584}	209.037	ar	96214.	93734.4	{93580., 93858.}	2.57718
48	R0	{1.82402, 10.0038}	{2.9884, 12.8326}	{{2.9884, 12.8326}, {2.9884, 12.8326}}	{63.8359, 28.2773}	inf	312.	7952.41	{4439., 12594.}	59.0896	0.17329	newly	2763.	3568.5	{1915., 5657.}	45.0129	0.145037	Reff	0.0676164	0.251778	{0.210533, 0.289307}	272.362	ar	97076.	93978.1	{93801., 94114.}	3.19116
49	R0	{1.6424, 11.1667}	{2.99939, 13.7076}	{{2.99939, 13.7076}, {2.99939, 13.7076}}	{82.6224, 22.7543}	inf	368.	4403.26	{2089., 6480.}	38.652	0.187761	newly	2454.	1854.32	{857., 2765.}	40.0991	0.226836	Reff	0.0891166	0.21291	{0.193731, 0.231853}	138.912	ar	95546.	94016.5	{93894., 94157.}	1.60083
50	R0	{2.05638, 14.3048}	{2.99939, 13.7076}	{{2.99939, 13.7076}, {2.99939, 13.7076}}	{45.8578, 4.17482}	inf	362.	7988.84	{4870., 12679.}	31.8875	0.158067	newly	3752.	3387.33	{2014., 5398.}	32.0881	0.152939	Reff	0.0564271	0.244357	{0.214036, 0.292081}	333.049	ar	98194.	94057.5	{93911., 94210.}	4.2126
